# A comprehensive review of probiotics and human health-current prospective and applications

**DOI:** 10.3389/fmicb.2024.1487641

**Published:** 2025-01-06

**Authors:** Bhutada Sarita, Dahikar Samadhan, Md Zakir Hassan, Elena G. Kovaleva

**Affiliations:** ^1^Department of Microbiology, Sanjivani Arts, Commerce and Science College, Kopargaon, India; ^2^Department of Technologies for Organic Synthesis, Institute of Chemical Technology, Ural Federal University named after the First President of Russia B. N. Yeltsin, Yekaterinburg, Russia; ^3^Bangladesh Livestock Research Institute, Savar, Bangladesh

**Keywords:** probiotics, human health, synbiotics, intestinal disorders and encapsulation, prebiotics

## Abstract

The beneficial properties of probiotics have always been a point of interest. Probiotics play a major role in maintaining the health of Gastrointestinal Tract (GIT), a healthy digestive system is responsible for modulating all other functions of the body. The effectiveness of probiotics can be enhanced by formulating them with prebiotics the formulation thus formed is referred to as synbiotics. It not only improves the viability and stability of probiotic cells, but also inhibits the growth of pathogenic strains. *Lactobacillus* and *Bifidobacterium* spp. are most commonly used as probiotics. The other microbial spp. that can be used as probiotics are *Bacillus, Streptococcus*, *Enterococcus*, and *Saccharomyces*. Probiotics can be used for the treatment of diabetes, obesity, inflammatory, cardiovascular, respiratory, Central nervous system disease (CNS) and digestive disorders. It is also essential to encapsulate live microorganisms that promote intestinal health. Encapsulation of probiotics safeguards them against risks during production, storage, and gastrointestinal transit. Heat, pressure, and oxidation eradicate probiotics and their protective qualities. Encapsulation of probiotics prolongs their viability, facilitates regulated release, reduces processing losses, and enables application in functional food products. Probiotics as microspheres produced through spray drying or coacervation. This technique regulates the release of gut probiotics and provides stress resistance. Natural encapsulating materials including sodium alginate, calcium chloride, gel beads and polysaccharide promoting safeguards in probiotics during the digestive process. However, several methods including, spray drying where liquid is atomized within a heated air chamber to evaporate moisture and produce dry particles that improves the efficacy and stability of probiotics. Additionally, encapsulating probiotics with prebiotics or vitamins enhance their efficacy. Probiotics enhance immune system efficacy by augmenting the generation of antibodies and immunological cells. It combats illnesses and enhances immunity. Recent studies indicate that probiotics may assist in the regulation of weight and blood glucose levels and influence metabolism and insulin sensitivity. Emerging research indicates that the “gut-brain axis” connects mental and gastrointestinal health. Probiotics may alleviate anxiety and depression via influencing neurotransmitter synthesis and inflammation. Investigations are underway about the dermatological advantages of probiotics that forecasting the onsite delivery of probiotics, encapsulation is an effective technique and requires more consideration from researchers. This review focuses on the applications of probiotics, prebiotics and synbiotics in the prevention and treatment of human health.

## 1 Introduction

### 1.1 Probiotics-a brief introduction

Probiotic is a Greek word meaning “for life,” coined by Lilley and Stillwell. Probiotics refer to microbes of non-pathogenic nature that are beneficial to their hosts (Soccol et al., 2014). Probiotics have been in use for quite a long time as Romans and Greeks, the ancient civilizations developed fermented milk and used it as probiotics, even the bible mentions this sour milk so the concept of probiotics is not entirely new (Hosono, 1992). Probiotics improve the microbial balance of the GI tract. World Health Organization (WHO) defines probiotics as “Live microbes which confer a health benefit to their host when administered in adequate amounts” (Chen and Sears, 2015). *Lactobacillus, Bifidobacterium, Enterococcus, Lactococcus*, and *Streptococcus* are most commonly used as probiotics (de Sequeira et al., 2022). Safety and functionality criteria must be cleared before using any microbial strain as a probiotic. These criteria include genetic stability, tolerance to acid and bile, ability to adhere to the gut lining, anti-genotoxic properties, non-pathogenic nature, production of lactic acid, tolerance to harsh processing conditions, and shorter generation time (de Melo Pereira et al., 2018). Probiotics exert their effects via enhancing the epithelial barrier, promoting microbial adherence to the intestinal mucosa while suppressing pathogen adhesion, modulating the immune system, and creating biochemicals that can suppress the growth of pathogenic microorganisms (Bermudez-Brito et al., 2012). These antimicrobial compounds are known as bacteriocins; they have active protein moiety. These bacteria also produce short chain fatty acids (SCFAs,) hydrogen peroxide (H_2_O_2_) and diacetyl, these biochemicals modify the intestinal microflora leading to positive health benefits (Hawrelak and BNat, 2013). Almost all strains of *Bifidobacteria* and *Lactobacilli* are capable of producing these bacteriocins.

### 1.2 Prebiotics and synbiotics

Prebiotics can be defined as indigestible food components that provide health benefits by restoring the growth of beneficial microbes in the gastrointestinal tract (GI)T. Prebiotics are known majorly for stimulating the activity and growth of good bacteria in the GIT. They stimulate the growth of bacteria present in the colon. Unlike other food components, they are hardly affected by hydrolyzing enzymes or acids which are present in our GIT, but are prone to fermentation by beneficial bacteria (Kuo, 2013). Jerusalem artichoke, chicory roots, berries, tomatoes, unrefined wheat, onions, asparagus, garlic, soybeans, undigestible carbohydrates, etc. are all sources of prebiotics (Pokusaeva et al., 2011). There are certain prebiotics that exhibits several health benefits apart from modulating the growth of beneficial microbes. They act as anti-inflammatory, anti-diarrheal and lower the risk of colon cancer (Peña, 2007). Alginate and agar that have been derived from seaweed. In addition to being abundant in the polysaccharide known as Ulvan, Gelidium is also a unique prebiotic for the bacteria known as *Faecalibacterium prausnitzii* (Saulnier et al., 2009). The by-products of their fermentation are short-chain chain fatty acids (SCFAs) namely acetic or propionic acids, these acids reduce the intestinal pH, but certain bacteria like *Bifidobacterium* are tolerant to SCFAs. This inhibits the growth of harmful bacteria and promotes the growth and development of beneficial bacteria like *Bifidobacterium* (Lacerda et al., 2020).

**Figure 1:**
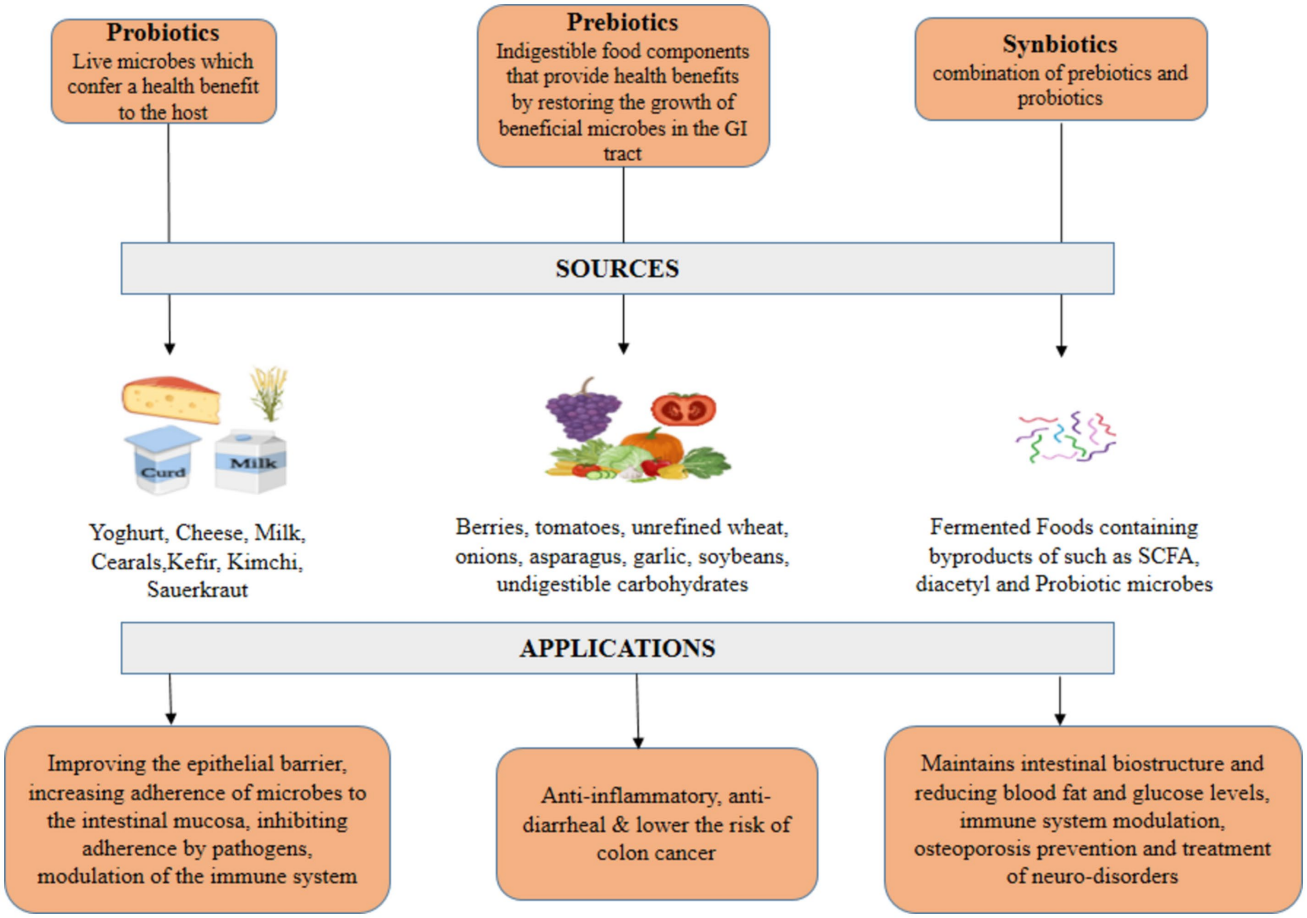
Sources and applications of probiotics, prebiotics and synbiotics.

Survival of probiotics is sometimes difficult in the GIT, to curb the survival difficulties synbiotics were created. The viability and efficiency of probiotics are dependent on several factors including oxygen, moisture, stress, pH, etc. (Mazziotta et al., 2023). Synbiotics are a combination of prebiotics and probiotics formulated in a way that not only improves the chances of survival of beneficial microbes, but also stimulates the growth and proliferation of the native bacterial population in the GI tract. Prebiotics improve the tolerance of probiotics to environmental factors like temperature, oxygenation and pH inside the GIT. But when prebiotics and probiotics are combined, efficiency of microbes and their tolerance to limiting factors get drastically improved causing a beneficial effect on the host’s body (Manigandan et al., 2012). Synbiotics not only inhibit the growth of harmful pathogens, but also maintains intestinal biostructure and reduce the undesired metabolite concentration. Synbiotics are quite efficient in reducing blood fat and glucose levels, immune system modulation, osteoporosis prevention and in the treatment of neuro disorders arising from abnormal hepatic functions. *Lactobacilli*, *B. coagulans*, *Bifidobacteria* sp., *S. boulardi*, etc. are the most commonly used probiotic strains in synbiotic formulations along with oligosaccharide-based prebiotics (Pandey et al., 2015). Synbiotics amalgamate probiotics (viable advantageous bacteria) with prebiotics (agents that facilitate their proliferation) to enhance health. Multiple factors influence synbiotic formulation. Choosing Probiotics and Prebiotics the selection of probiotic and prebiotic strains is essential. Probiotics must be selected for their health benefits and capacity to last in the gastrointestinal tract, whereas prebiotics should enhance their proliferation. The chosen strains and substrates must collaborate to attain health benefits (Chan and Liu, 2022). Sustainability ensuring probiotic viability during manufacturing, storage, and distribution poses significant challenges. The survival of probiotics in yogurt is influenced by pH, moisture, and oxygen concentrations. Consequently, probiotics are frequently encapsulated to shield them from environmental stressors and maintain their stability until they arrive in the stomach. The matrix formulation of probiotics and prebiotics influences the release and effectiveness of synbiotic products. The formulation must deliver probiotics at the appropriate time and location inside the gastrointestinal tract to optimize health benefits (Chan et al., 2021; Chan and Liu, 2022). Synbiotics may be classified into several categories, such as dietary supplements and functional foods, complicating regulation. The diversity may influence the approval and market introduction of synbiotic products. Synbiotics are increasing in popularity; yet, consumers are still acquiring knowledge regarding their advantages and distinctions from probiotics and prebiotics. The market applications of synbiotics are expanding as consumers become increasingly informed about gut health, probiotics, and prebiotics. Primary applications encompass Synbiotics are commonly used into yogurts, smoothies, and dietary supplements (Rashidinejad et al., 2022). These products provide convenient solutions for intestinal health. Moreover, Synbiotics may address dysbiosis-related conditions such as gastrointestinal disorders, obesity, and metabolic syndrome. Additionally, microbiome research may provide tailored synbiotic formulations to address specific health requirements and microbiome characteristics. This could enhance the effectiveness of synbiotics for particular health outcomes (Chaturvedi and Chakraborty, 2021) (Figure 1).

#### 1.2.1 Safety criteria of probiotics, potential risks and safety concerns for specific populations

The safety of probiotics is crucial due to their consumption by diverse populations, including infants, the elderly, and immunocompromised individuals. The subsequent safety criteria are typically evaluated. Incorporating with Probiotic strains must be accurately characterized to ensure identification, purity, and viability (Mazziotta et al., 2023). This encompasses verifying the strain’s viability and determining its genetic and phenotypic characteristics. Consumer safety necessitates clearly defined strains with established safety attributes. Probiotics must undergo evaluation for pathogenicity (Damián et al., 2022). Therefore, evaluation of strains can induce diseases or generate toxic substances. Animal models are employed to investigate safety hazards such as endocarditis in vulnerable populations. Probiotic products must undergo testing for harmful bacteria, mycotoxins, and heavy metals. The final product must be free from hazardous contaminants to ensure customer safety. Clinical Safety—Probiotics must demonstrate safety in the target population as evidenced by clinical research. This entails overseeing trial adverse effects and confirming the safety of probiotic strains. Regulations differ by region, although probiotic products must comply. This entails adhering to the safety and efficacy requirements for probiotics established by health authorities for food and supplements (Tegegne and Kebede, 2022).

Quality Control—Manufacturers must rigorously oversee quality during the whole production process. This entails maintaining the potency, stability, and shelf life of probiotic products while adhering to superior manufacturing practices. Adverse Events—Post-marketing surveillance is essential to monitor probiotic side effects (Bodke and Jogdand, 2022). This ongoing examination highlights potential safety concerns that may arise post-release of items. At-risk populations, including individuals with compromised immune systems or other health conditions, should be prioritized to mitigate risks. The safety of probiotics is crucial as they are consumed by diverse populations, including infants, the elderly, and immunocompromised individuals (Sharifi-Rad et al., 2020). The subsequent safety criteria are typically evaluated. Probiotic strains must be accurately characterized to ensure identification, purity, and viability. This entails verifying the strain’s viability and characterizing its genetic and phenotypic attributes. Consumer safety necessitates well delineated strains with established safety attributes. Probiotics must undergo testing for pathogenicity. We evaluate if strains can induce diseases or generate toxic substances. Animal models are utilized to investigate safety hazards such as endocarditis in vulnerable populations. Probiotic products must undergo testing for harmful bacteria, mycotoxins, and heavy metals. The final product must be free of hazardous contaminants to ensure customer safety. Clinical Safety—Clinical trials must establish the safety of probiotics within the target population. This entails overseeing trial adverse effects and confirming the safety of probiotic strains. Regulatory compliance: Standards for probiotic products vary by region. This entails adhering to the safety and efficacy requirements for probiotics established by health authorities for food and supplements (Victoria Obayomi et al., 2024).

Manufacturers must rigorously enforce quality control measures during production. This include maintaining the potency, stability, and shelf life of probiotic products while adhering to superior manufacturing practices. Adverse Events—Post-marketing surveillance is essential for monitoring probiotic side effects. This ongoing examination detects safety concerns that may arise post-release of items. Priority should be given to vulnerable groups, including individuals with compromised immune systems or other health conditions. In the United States, probiotics are regulated by the Food and Drug Administration (FDA) as dietary supplements or food products (Al-Rashidi et al., 2022). The 1994 Dietary Supplement Health and Education Act (DSHEA) is applicable; however, pre-market approval is not mandated. Manufacturers are required to label and guarantee product safety. Opportunistic infections, particularly in immunocompromised individuals, afflict probiotics. Probiotic strains and other benign bacteria can induce illness when the immune system is compromised. Users of central venous catheters and those with severe diseases have experienced *Saccharomyces fungemia* (Palacios et al., 2023). Some individuals may encounter bloating, flatulence, or diarrhea after initiating probiotic use. The symptoms are often mild and temporary; however, consumers express concern. Probiotics may influence the efficacy of immunosuppressive medications. This combination may elevate the risk of infection or diminish treatment efficacy. Probiotics can be detrimental to people undergoing chemotherapy, organ transplants, and those with HIV/AIDS. Individuals with diminished infection resistance require an assessment of probiotic strain safety prior to utilization. Administer probiotics wisely to avert gastrointestinal complications in neonates and early children (Ashaolu, 2020).

## 2 Probiotics classification

At present, there are a variety of microbes that are being used as probiotics (Hawrelak and BNat, 2013). Table 1 represents bacterial strains that are commonly used as probiotics. Bacteria belonging to the *Lactobacillus* genus are gram-positive bacilli that are capable of producing lactic acid in the GIT and GUT (Genitourinary tract), they are anaerobes that can improve uptake and bioavailability of minerals and reduce intestinal permeability. In fact, some strains of this genus exhibit anti-cancer and hypolipidemic activity (Cichonska and Ziarno, 2022).

**Table 1:** Bacterial strains that are commonly used as probiotics.

Genus	Species
*Lactobacillus* spp.	*acidophilus* *rhamnosus* *fermentum* *johnsonii* *lactis* *reuteri*
*Bifidobacterium* spp.	*breve* *infantis* *longum* *bifidum* *lactis* *thermophilum*
*Bacillus* spp.	*coagulans*
*Streptococcus* spp.	*thermophilus*
*Enterococcus* spp.	*faecium*
*Saccharomyces* spp.	*cerevisae*

*Bifidobacterium* is pleomorphic, anaerobic, gram-positive bacilli which produce acetic acid and lactic acid as metabolic by-products. It can reduce the effects of *Helicobacter pylori* infection if used in combination with either *Saccharomyces cerevisiae* and *Lactobacilli* (Chen et al., 2019).

*Bacillus coagulans* are known for producing lactic acid and are sometimes commercialized as *Lactobacillus sporogenes*, but it is not a part of normal intestinal flora, unlike *Lactobacillus.* But it is quite helpful in inhibiting colonization by pathogens and restoring normal intestinal microbiota and they are highly prone to acidic environments and high temperatures (Cao et al., 2020).

*Saccharomyces cerevisiae* or *S. boulardii* is commonly used for the treatment of diarrhea, in natural forms, it is isolated from skin of tropical fruits of Indochina. It is quite resistant to acid and temperature stress, thus can be easily used as a probiotic (Bhukya et al., 2019).

Probiotics refer to living microorganisms that provide a range of health benefits when ingested or administered for optimal levels within the body. Table 1 provides information on the prevalent probiotic bacteria present in yogurt, fermented meals, dietary supplements, and beauty items (Elhossiny et al., 2023).

### 2.1 Lactobacillus acidophilus

*Lactobacillus acidophilus* is a strain of probiotic bacteria that has been widely utilized due to its potential health advantages. This strain possesses the capacity to cling to diverse intestinal cells, exhibits tolerance to bile, and demonstrates resistance to acid, which are essential attributes for a probiotic strain. Nevertheless, previous research conducted in laboratory settings has demonstrated that certain strains of *Lactobacillus acidophilus* had the ability to decrease cholesterol levels by over 50%. This finding underscores their potential significance in enhancing cardiovascular well-being, particularly when used in conjunction with other probiotic strains. Moreover, it has demonstrated efficacy in the prevention of gastrointestinal illnesses among adults and the mitigation of symptoms associated with the common cold in children. The strains of *Lactobacillus acidophilus* that are commercially accessible include LA-1, LA-5, NCFM, DDS-1, and SBT-2026 (Elhossiny et al., 2023).

### 2.2 Lactobacillus rhamnosus

The strain *Lactobacillus rhamnosus* has developed distinctive adaptations that enable its survival in the acidic and basic environments present in the human body. The capacity of *L. rhamnosus* to attach and colonize the intestinal walls enables it to potentially provide extended advantages. Consequently, it is frequently used into yogurts, cheeses, milk, and other dairy products to augment probiotic levels. Additionally*, L. rhamnosus* plays a pivotal part in the process of cheese ripening, so boosting the overall flavor. Moreover, some strains of *L. rhamnosus* have demonstrated advantageous effects on both adults and children, specifically in the treatment of irritable bowel syndrome (IBS), eczema, allergies, and immune system support (Gao et al., 2022).

### 2.3 Lactobacillus fermentum

*Lactobacillus fermentum* is a probiotic bacterium that exhibits abilities in adhesion and anti-infective properties. It is commonly found in cheese ripening and is classified as a non-starter lactic acid bacteria (NSLAB) within specific cheese varieties. These characteristics suggest that *Lactobacillus fermentum* has the potential to effectively combat infections and promote health in the urinary-reproductive tract. In addition, it has been observed that *Lactobacillus fermentum* strain JDFM216 has the potential to enhance cognitive and physiological functioning, while also demonstrating immunomodulatory properties. This particular strain has been linked to heightened phagocytic activity of macrophages, elevated synthesis of IgA, and heightened activation of immune cells. The probiotic strain *Lactobacillus fermentum* has been recognized for its antibacterial and antioxidative capabilities, ability to metabolize cholesterol, and possible contribution to cardiovascular health. These attributes make it a distinctive strain with prospects for boosting health. Furthermore, it has been observed that *Lactobacillus fermentum* strains exhibit a notable capacity for auto-aggregation, a crucial factor in their ability to cling to epithelial cells and facilitate the production of biofilms within the gastrointestinal tract (Anjum et al., 2014).

### 2.4 Lactobacillus johnsonii

One of the initial cultures suggested as a probiotic dairy supplement is *Lactobacillus johnsonii* strain LA-1. This strain is utilized in Nestlé’s LC-1 yogurt products and possesses the capacity to augment immune responses, withstand diverse conditions such as bile salts and antibiotics, combat antimicrobial multidrug resistance microorganisms, and maintain a high level of probiotic viability in food items. Moreover, *Lactobacillus johnsonii* has demonstrated the ability to decrease the adhesion and activity of pathogenic strains, impede the growth of gut pathogens, and shorten the duration of diarrhea and enterocolitis (Tavasoli et al., 2022).

### 2.5 Lactobacillus lactis

The lactic acid bacterium species known as *Lactobacillus lactis* has been the subject of substantial research due to its potential probiotic capabilities in enhancing immune system function and alleviating inflammatory bowel illness. This analysis presents a comprehensive examination of the probiotic properties and potential health advantages associated with *Lactobacillus lactis*, which is commonly employed as a probiotic. The evaluation is based on specific criteria including bile tolerance, acid resistance, cholesterol assimilation activity, and adhesion to intestinal cells. The anti-inflammatory capabilities of some strains of *Lactobacillus lactis*, such as *L. lactis* ML-2018, have been recognized, particularly in their ability to inhibit the production of inflammatory factors triggered by lipopolysaccharides (LPS). Certain strains of *Lactobacillus lactis* have been linked to enhancing the immune system and avoiding infections in the gastrointestinal and upper respiratory tract (Zanjani et al., 2017; Nazia et al., 2014).

### 2.6 Lactobacillus reuteri

*Lactobacillus reuteri* has been linked to a wide range of health advantages, encompassing the prevention and management of urogenital disorders and bacterial vaginosis in females, atopic disorders, food hypersensitivity, and the prevention of dental caries. Furthermore, it has been examined for its capacity to prevent colitis and decrease contacts between P-selectin-associated leukocytes and platelets with endothelial cells, emphasizing its significance in intestinal illnesses. Extensive research has been conducted on its capacity to inhibit the growth of harmful bacteria, yeasts, and other microorganisms, so demonstrating its potential as a helpful probiotic for the treatment of gastrointestinal and urogenital ailments, including infantile colic (Assimos, 2020).

### 2.7 Bifidobacterium breve

The bacterial species *Bifidobacterium breve*, belonging to the genus *Bifidobacterium*, is renowned for its probiotic characteristics. This bacterium is a symbiotic organism that resides in the human intestines and has been employed for the treatment of several ailments, such as constipation, diarrhea, irritable bowel syndrome, and even the common cold and flu. Scientific research has substantiated several applications, showcasing the potential physiological advantages of *B. breve*. This rod-shaped bacterium is gram-positive, anaerobic, and non-motile. It creates branches with its neighboring organisms. *Bifidobacterium breve* strains have been extensively employed in the field of pediatrics and are recognized as the prevailing species in the gastrointestinal tract of infants who are breastfed. Furthermore, they have been extracted from human milk, emphasizing their inherent existence in the gastrointestinal tract of infants. The strains of *B. breve* have undergone successful trials in pediatric populations and have demonstrated efficacy in addressing various health issues, hence establishing their significance as a probiotic strain for enhancing digestion and general well-being (Zanjani et al., 2017).

### 2.8 Bifidobacterium infantis

*Bifidobacterium infantis*, scientifically referred to as *Bifidobacterium longum* subsp. *infantis*, is a benign bacterial strain that naturally inhabits the mouth and digestive system. It belongs to the same group as *Lactobacillus* and is a type of lactic acid bacteria that is essential for keeping a healthy digestive system. *Bifidobacterium infantis* 35,624 has been extensively studied and has been specifically examined for its potential in treating irritable bowel syndrome (IBS). Its efficacy in alleviating symptoms such as bloating, bowel issues, pain, and gut dysbiosis in infants has been demonstrated. Moreover, it has been linked to enhanced management of gastrointestinal distress and enhancement of the overall well-being of pediatric patients diagnosed with irritable bowel syndrome (IBS) (Azad et al., 2018; Sanders et al., 2018).

### 2.9 Bifidobacterium longum

*Bifidobacterium longum* is a commensal bacteria that resides in the gastrointestinal system and is widely acknowledged as a prominent constituent of the human gut microbiota. It is particularly prevalent in the baby gut, where it is the most numerous species. It demonstrates a multitude of advantageous health benefits, encompassing the synthesis of bioactive compounds and the interaction between bifidobacterial surface-associated molecules and the host organism. Extensive study has been conducted on *Bifidobacterium longum*, revealing its effectiveness in managing the symptoms associated with irritable bowel syndrome (IBS), such as bloating, diarrhea, abdominal pain, and distress. Furthermore, it has been examined for its capacity to prevent antibiotic-associated diarrhea in children and irritable bowel syndrome (IBS). Additionally, it has been explored for its effectiveness in promoting remission in active ulcerative colitis and its ability to degrade the mucin layers in the host gut, thereby preserving the microbial community. Moreover, much research has been conducted on the phytochemical bio-catalytic properties of this substance, its capacity to adhere to cells, its potential to inhibit carcinogenesis in cell lines, its ability to modulate immune cells, and its potential to reduce allergic reactions in mice models and treat inflammatory bowel disease (Azad et al., 2018).

### 2.10 Bifidobacterium lactis

*In vitro* testing has extensively established the strain characteristics and processes of *Bifidobacterium lactis*, demonstrating its great stability in meals and freeze-dried powders. Clinical evidence has demonstrated that *Bifidobacterium lactis* HN019 promotes gut health, digestion, and immunological function (Sanders et al., 2019).

### 2.11 Bifidobacterium thermophilum

*Bifidobacterium thermophilum* is acknowledged as an aerotolerant bacteria, possessing the ability to endure and flourish in oxygen-depleted settings, rendering it a promising contender for probiotic application. *Bifidobacterium thermophilum* demonstrates bacteriocin-like antimicrobial properties against various pathogens, including *Listeria* spp., *Salmonella* spp., *Campylobacter jejuni* in broiler and rota virus infection. This makes it a highly promising candidate for incorporation into probiotic formulations and functional foods due to its aerotolerance and antimicrobial activity (Sanders et al., 2019).

### 2.12 Bacillus coagulans

*Bacillus coagulans* is a spore-forming probiotic bacteria renowned for its exceptional resilience to adverse settings and its multitude of probiotic traits, enabling it to remain inactive under harsh conditions, such as elevated gastric acidity. The inherent durability of this substance allows it to endure harsh conditions, rendering it highly efficient in mitigating gastrointestinal distress and various other medical conditions. Additionally, it has the ability to control the host’s symbiotic microbiota and hinder the proliferation of harmful bacteria, so promoting general gastrointestinal well-being and providing support to the digestive and immune systems. In natural food sources, such as fermented foods like sauerkraut, kimchi, and yogurt, *Bacillus coagulans* can be found. Furthermore, it finds utility in several probiotic food additives, showcasing its suitability for industrial implementation in the food sector (Ma et al., 2021).

### 2.13 Streptococcus thermophilus

The bacterium *Streptococcus thermophilus* is frequently employed in the manufacturing of many dairy commodities, such as cheeses and yogurt. It aids in the hydrolysis of lactose in milk, leading to the distinctive flavor and consistency of yogurt. Furthermore, this substance is acknowledged for its capacity to decrease the fat content in specific types of cheeses, such as Swiss cheese, by means of generating natural polymer extracts. This particular strain of probiotics has been linked to a range of health advantages, such as bolstering the immune system and mitigating inflammation in the gastrointestinal and urogenital systems. Additionally, it has demonstrated potential in combating viral, fungal, and parasitic infections. The coexistence of *Bifidobacterium bifidum* and *Streptococcus thermophilus* in babies has been associated with a reduced incidence of rotavirus diarrhea. This combination has been found to potentially mitigate inflammation-induced harm resulting from sepsis, hence underscoring its potential as a dietary supplement (Qu et al., 2023).

### 2.14 Enterococcus faecium

There is no evidence to suggest that probiotic strains of *Enterococcus faecium* possess the capacity to induce antibiotic resistance. In order to guarantee the safe consumption of probiotic products containing *Enterococcus faecium*, stringent safety standards have been established to exclusively employ microbial strains that are deemed suitable for use in food or food supplements. It possesses a distinct advantage in enduring the process of digestion and thriving in the gastrointestinal tract, fostering a harmonious gut milieu by engaging in competition with detrimental species for nutrients and adhesion sites. Moreover, strains of *Enterococcus faecium* exhibit promising therapeutic properties, including the ability to prevent and treat diarrhea in domesticated animals, as well as block the proliferation of pathogenic *Listeria* spp. (Tilwani et al., 2022).

### 2.15 Saccharomyces cerevisiae

*Saccharomyces cerevisiae*, specifically the variant *S. boulardii*, is widely acknowledged for its probiotic capabilities and has undergone extensive investigation for its advantageous impacts on gastrointestinal well-being in both human and animal populations. It is commonly employed as an adjunctive measure against gastrointestinal tract disorders, including inflammatory bowel disease, and for the management of diverse forms of diarrhea. The defensive mechanisms of this entity are manifested through the binding and neutralization of enteric pathogens or their toxins, the reduction of inflammation, and the stimulation of IgA secretion. These strains possess the potential to be employed in functional food applications and exhibit the capability to safeguard DNA from harm. The possible probiotic features of *Saccharomyces cerevisiae* yeast include its tendency to auto-aggregate, co-aggregation with pathogens, hydrophobicity, ability to survive in simulated gastrointestinal tract settings, and its ability to adhere to Caco-2 cells. These aforementioned attributes render them viable contenders for therapeutic implementations (Fernandez-Pacheco et al., 2018).

## 3 Substrates for probiotics

### 3.1 Cereals as a substrate for probiotics

In Asia and Africa, lactic fermentation of grains is a processing method to produce various products such as drinks, porridge and *amaj*. During fermentation, acidity increases due to microbial activity and the accumulation of lactic acid and other organic acids. On the other hand, in western countries, grains such as wheat and rye are used to produce sour dough. *Lactobacillus* and *Bifidobacteria* have different nutritional requirements, including the need for carbohydrates, amino acids, peptides, fatty esters, salt, and acid derivatives (Setta et al., 2020). It is nucleic and vitamin. The composition of the main carbohydrates of cereals includes starch, dietary fiber soluble and insoluble in water, several Sugar includes glucose, glycerol, stachyose, xylose, fructose, maltose, sucrose and arabinose. Compared to milk, cereals contain It is higher than some essential vitamins, dietary fiber and minerals, especially phosphorus. In a study of microbial species with human source includes *Lactobacillus plantarum, Lactobacillus acidophilus, Lactobacillus rotoi*, and *Lactobacillus fermentum*. It was isolated and grown in the culture medium containing grains such as malt, barley and wheat (Behera et al., 2018).

### 3.2 Dietary fiber obtained from cereals and its prebiotic effect

Edible fiber or carbohydrate of plants is a dietary fiber that is resistant to hydrolysis by digestive enzymes and is divided into two categories soluble and insoluble fiber is divided. Soluble fiber includes non-starch polysaccharides, which, due to the creation of a viscous environment, It delays the emptying time of the stomach and reduces the absorption of glucose and sterol by the small intestine. Insoluble fiber included Lignin, cellulose and hemicellulose. The amount of dietary fibers in cereals decreases from the external pericarp side to the endosperm; Except Arabinoxylan, which is the main component of the endosperm cell wall, and conventional milling methods have been developed for the separation and purification of dietary fiber (Seyedain-Ardabili et al., 2016; Brasil et al., 2011).

### 3.3 Vegetables and fruits as substrates for probiotics

Using probiotic microorganisms in vegetable-based substrates has been shown to provide numerous health benefits. Raw and fermented vegetables are substrates for probiotic bacteria because they contain nutrients they can easily absorb. Microbial populations can also be hosted and delivered via plant-based matrices due to their high nutritional, fiber, vitamin, mineral, and bioactive phytochemical content. In novel vegetable probiotic products, *Lactobacillus acidophilus, Lactobacillus casei, Lactobacillus plantarum, Lactobacillus rhamnosus*, and *Bifidobacterium lactis* are commonly used. Vegetables include probiotic microorganisms that stimulate the immune system, prevent gastrointestinal illnesses, and regulate fat storage. They also regulate gut microbial composition and metabolism (Palanisamy et al., 2024). Vegetables contain probiotic bacteria, a natural source. Fermented foods, particularly raw and fermented vegetables, contain *Lactobacillus acidophilus*, a gut bacteria. Fresh produce like fruits and vegetables contains a variety of germs, some of which may be healthy. The average apple contains 100 million microorganisms, many of which are harmless or useful. Fruits, vegetables, and cereals may carry probiotics. Fruit and vegetable juices and raw and fermented vegetables have been employed as probiotic bacteria substrates due to their nutrient content. *Lactobacillus plantarum,* a vegetable-fermented lactic acid bacterium, has been studied for its probiotic qualities. This strain was immunomodulating and antibacterial against pathogenic bacteria, suggesting it could be a probiotic and food additive (Gagnière et al., 2016). White peas, green peas, chickpeas and dragon fruit have lactic acid bacteria. Lactic acid bacteria (LAB) including *Lactobacillus, Enterococcus*, and *Bifidobacterium* are present in vegetable substrates (Jha et al., 2022). When added to vegetable substrates through lactic fermentation, these probiotic bacteria regulate the gut flora and improve health. However, using vegetable substrates to transport probiotic lactic acid bacteria (LAB) in food matrices. Micronutrients, antioxidants, and fiber make vegetable substrates ideal for bioprocess development (Sharma et al., 2021). Fruits, vegetables, legumes, and cereals have been studied as probiotic vehicles, demonstrating their gut health benefits. Fruits and vegetables can feed probiotic microorganisms in lactic fermentation. Among them are mango, apple, banana, passion fruit, carrot, orange, and soybeans. Probiotic bacteria can grow and thrive on these veggies’ various nutritional profiles and bioactive substances. Probiotic bacteria are found in kimchi and sauerkraut. Kimchi contains *Leuconostoc*, *Weissella*, and *Lactobacillus*, while sauerkraut contains *Leuconostoc mesenteroides, Lactobacillus plantarum, Pediococcus pentosaceus*, and others (Junnarkar et al., 2019). These microorganisms may aid digestion. The best thing for gut health is eating a range of natural meals, particularly fresh produce. Fruits and vegetables contain more microbial species than probiotic pills. Thus, eating a variety of fresh fruits and vegetables supports gut microbiota health and general wellness. Fermented fruits and vegetables contain *Lactobacillus*, *Streptococcus*, *Leuconostoc*, and other LAB. These bacteria dominate fermented foods and may be probiotic. LAB, along with other bacteria, yeast, and fungi, ferment these foods and generate live microorganisms, making them good carriers for probiotic bacteria (Bernal-Castro et al., 2024). Probiotic bacteria are found in kimchi and sauerkraut. Kimchi contains *Lactobacillus kimchii* and other lactic acid bacteria, which may aid digestion (Acevedo-Martínez et al., 2018). *Leuconostoc mesenteroides, Lactobacillus plantarum, Pediococcus pentosaceus,* and others in sauerkraut may enhance gut health. Fruits include vitamins, minerals, carbohydrates, fibers, and antioxidants, making them good probiotic substrates. Fruit surfaces’ microarchitecture protects probiotic bacteria from stomach acid, enhancing their survival and health benefits. Fruit juices, especially citrus, are promising probiotic carriers. Fruit juices are ideal for probiotic bacteria growth because lactic acid bacteria can ferment mildly acidic plant and vegetable substrates. The research utilized fruits as raw materials for the growth of *Lactobacillus acidophilus* and *Lactobacillus plantarum*, two probiotic bacteria often found in fruit substrates (Xia, 2020). These fruits were ideal substrates for non-dairy probiotic products because they offered the essential conditions for probiotic bacteria growth and lowered pH and enhanced vitality after cold storage at 4°C. *Lactobacillus acidophilus* and *Lactobacillus plantarum* are common probiotic bacteria in fruits and non-dairy probiotic beverages. These lactic acid bacteria strains survive on fruit substrates because they tolerate acidity. Probiotic microorganisms utilized in apple, banana, carrot, and tomato juices to make non-dairy probiotic drinks. Dairy products are a good source of probiotics, which promote health. However, lactose intolerance and high cholesterol in dairy products have increased demand for non-dairy probiotics (Bernal-Castro et al., 2024).

## 4 Oligosaccharides compounds and application in prebiotic and probiotic

Biological processing of cereals using enzymatic reactions or through fermentation causes the production of a high amount of oligosaccharides with potential properties. It becomes a prebiotic. These oligosaccharides are isolated from plant materials or they are made enzymatically. Oligosaccharides such as lactose, fructo-oligosaccharides and transgalacto-oligosaccharides stimulate the growth of probiotics. In the food industry, these compounds are added as supplements to some baby products, which have properties similar to Oligosaccharides of human milk. Resistant starch is usually produced through partial hydrolysis with acid, heat treatment, extrusion cooking, chemical modification and Polymerization takes place again. As a useful fiber, resistant starch has an important role in the physiology of digestion and the like Oligosaccharides are indigestible. This combination provides fermentable carbohydrates for river bacteria. Including the beneficial properties of resistant starch include the production of desirable metabolites in the intestine, including short-chain fatty acids, and as a prebiotic, it reduces the risk of gastrointestinal diseases. In addition to therapeutic effects, this type of starch improves appearance, texture, and feel compared to other conventional fibers (Hosseinvand et al., 2022).

## 5 Encapsulation method for onsite delivery of probiotics

Complete efficiency of any probiotic can be achieved only if the number of viable organisms is either higher or is equal to 10^7^ CFU/mL (Serna-Cock and Vallejo-Castillo, 2013). There are several other factors that affect the survival of microbe that is being used as probiotics. These factors include H_2_O_2_ production, pH, temperature, presence of bile salts and acids, etc. All these factors determine the efficiency and viability of probiotics. Providing a shield against these factors is quite an interesting approach these days. This approach enables delivery at the exact site of action which not only improves the viability of organisms used in formulation but also enhances their stability (Alvarez et al., 2021). With advancements in nanotechnology, numerous nanocarriers are available which can be conveniently used as carriers for delivering probiotics (Kumari et al., 2014).

Encapsulation can be defined as continuously coating any active agent to protect it from the external environment. A number of materials are being used for the encapsulation of drugs but probiotics are living cells so the choice of encapsulating material has to be specific as the polymer used must be biocompatible and biodegradable. The material used for encapsulation must allow bi-directional transport of nutrients in order for probiotic cells to survive (Gurruchaga et al., 2015). The effectiveness of encapsulation is dependent on the matrix that is utilized, which can be made up of either substances that are synthetic or substances that are natural. Materials such as alginate, carrageenan, whey protein, gelatin, chitosan, cellulose acetate phthalate, and locust bean gum are utilized widely in the process of microencapsulation. Within the temperature range of 60–80°C and alginate maintains its stability and has the potential to maintain cell viability in acidic environments (Bollinger et al., 2024).

Encapsulation techniques are crucial for safeguarding probiotics and augmenting their viability (Puscaselu et al., 2020). This document presents a comparison of frequently employed techniques. The spray drying method is an efficient and widely employed process in the food industry, particularly for improving the biofortification of fruit juice. Conversely, the elevated temperatures associated with spray drying may harm the probiotics. Co-encapsulation technology refers to the simultaneous encapsulation of many components. It is favored over encapsulating individual components because it is more convenient and cost-effective (Ansari et al., 2023). Furthermore, it has been ascertained that co-encapsulation enhances long-term preservation efficacy, resulting in its extensive application within the pharmaceutical industry. The extrusion method is a mild approach for encapsulating probiotics, characterized by its simplicity and cost-effectiveness, while minimizing cellular damage. A considerable quantity of probiotics remains encapsulated via extrusion and alginate techniques. Emulsion methodologies, often known as two-phase system approaches, are a fundamental strategy for encapsulating probiotic microorganisms (Zhang et al., 2023). The specifics of the methodology may vary, but it generally involves formulating an emulsion of the probiotic substance inside a suitable carrier material for the intended application. Probiotic encapsulation technology, referred to as PET, has shown promising results in both preclinical and clinical studies, resulting in the incorporation of probiotics into various products. Nonetheless, despite encapsulation, ensuring the sustained viability of cells for prolonged durations remains challenging. Pharmaceuticals and nutraceuticals available in European pharmacies and supermarkets frequently use probiotic components (Cui et al., 2021). However, encapsulated probiotics are utilized in numerous nutritional supplements. This is implemented to guarantee that the bacteria can endure the manufacturing process, subsequent storage, and transit through the digestive system. Encapsulated probiotics are employed in several food and beverages items, including yogurts, cheeses, and fermented beverages, among others. The encapsulation enhances the product’s shelf life and safeguards the probiotics from harsh manufacturing conditions. Pharmaceuticals that incorporate probiotics through the management of specific health conditions, such as gastrointestinal disorders, can be achieved by the use of encapsulated probiotics in particular pharmaceutical formulations. The encapsulation enables probiotics to reach specific regions of the body (Palacios et al., 2023). Carrageenan apart from being used as a feed additive can also be used as potential material for encapsulation. Since it is approved both by FDA and joint (Food Agricultural Organization) FAO/WHO. It forms a gel that can be used for encapsulation of cells, this gel however hardens at room temperature. This also enhances the stability of probiotics (Chakraborty, 2017).

Whey proteins are amphoteric in nature; this enables their mixing with carrageenan and pectin. Their net charge becomes positive when pH drops below isoelectric point as a result of which they can interact with negatively charged polysaccharides. Thus, they can be effectively used as immobilization material (Vonasek et al., 2014).

Deacetylation of chitin yields a positively charged polysaccharide chitosan. When pH is higher than 5.4 it becomes insoluble in the media, this is one of its major limitations as in case of high pH the contents are not fully released in the gut this limits the effectiveness of probiotics. On the contrary CAP (Cellulose Acetate Phthalate) is insoluble if pH is below 5, but it does not form beads like Chitosan, it can however be used as a coating material to further improve the stability of material used in encapsulation (Rokka and Rantamäki, 2010).

At present either extrusion or emulsification technique is used for the encapsulation of microbial cells. The former one is a quite simple technique as it can be automated, it involves retention of a large number of cells and it yields beads which are basically gelled droplets. Emulsification technique yields capsules that are either oily or aqueous droplets (Frakolaki et al., 2021).

Microencapsulation is a protective method of compounds to avoid deterioration due to environmental factors such as oxygen, temperature, moisture, enzymes, and acids; it also allows the delivery of encapsulated compounds in a specific site. Various biopolymers are used as wall materials for bioactive components incorporated into foods (Altamirano-Fortoul et al., 2012).

In recent years, various microencapsulation techniques using cereal components have been used to improve the viability of probiotic strains in Extra-beneficial products are used. The possibility of using corn starch granules containing high amylose as a system delivery was investigated for probiotic bacteria (Hosseinvand et al., 2022). In this review, Bifidobacterium strains isolated from humans starch granules were glued. Laboratory studies showed an increase in the survival of probiotic breeds in these conditions. The use of calcium alginate for microencapsulation of probiotic bacteria in yogurt was done by researchers. The binding of corn starch (prebiotic) with alginate also improved the microcovering of living bacteria. Methods such as spray drying for production the microbial microbeads that are uniformly coated and contain live bacteria are the focus of many researchers (Krasaekoopt et al., 2004).

## 6 Role of probiotics in promoting human health

Probiotics are used for the management of an array of disorders and unusual physiological conditions. There is enough evidence indicating the potential of various probiotic strains in the treatment of a number of health issues including gastrointestinal issues, tumors, respiratory issues, etc. The swift advancement of microbiome science has resulted in various applications of probiotics and prebiotics. Synbiotics, a combination of probiotics and prebiotics, are being formulated to enhance their efficacy. Probiotics are now under investigation for their potential in treating obesity, metabolic syndrome, respiratory infections, and COVID-19. Probiotics and prebiotics are projected to reach approximately $50 billion in sales and manufacturing within a few years (Bodke and Jogdand, 2022). This increase signifies consumer knowledge of gastrointestinal health and its impact on overall well-being. As the microbiome is comprehended more thoroughly, these products may enhance neurological and cancer preventive results. Recent studies on probiotics and prebiotics are uncovering their potential roles in neurobiology and cancer prevention. The search results do not address probiotics and prebiotics in neurobiology; nonetheless, the gut-brain axis is receiving increased attention in research. This study indicates that gut microbiota may influence brain function and behavior, potentially impacting depression, anxiety, and neurodegenerative diseases. Probiotics and prebiotics influence gut microbiota, perhaps benefiting neurological health. Investigations into probiotics and prebiotics for cancer prophylaxis appear encouraging (The Lancet Global Health, 2020). Numerous illnesses, including cancer, are associated with gut microbial dysbiosis. Prebiotics and probiotics may restore equilibrium and enhance immune surveillance, potentially exhibiting oncostatic properties. Numerous epidemiological and experimental studies have enhanced our understanding of probiotics and microbial therapy as anticarcinogenic agents (Cui et al., 2021). Certain human studies indicate that probiotics and prebiotics are beneficial. Certain clinical probiotic strains may decrease postoperative inflammation in cancer patients. Oral probiotics alleviated diarrhea associated with chemotherapy or radiotherapy. Probiotic aerosol therapy is an innovative treatment for preventing lung metastases in high-risk melanoma patients. Altered gut microbiota influences cancer progression and the efficacy of anticancer therapies. *In vivo* and molecular studies on probiotics and cancer prevention have demonstrated encouraging outcomes (Ezeji et al., 2021).

### 6.1 Probiotics in cancer treatment

Cancer is undoubtedly one of the leading causes of death around the globe. Nearly 755 cancer patients die as a result of diet and lifestyle-associated factors and the diet-related factors alone account for 50% of deaths. Numerous *in-vitro* and animal studies indicate the role of intestinal and gut microbiota in reducing the risk of death from diet-related factors. Probiotics play an important role in reducing the risk of colon and bladder cancer. *Helicobacter, Pseudomonas and Acinetobacter* are responsible for tumor formation in the colon which ultimately leads to colon cancer. They proliferate easily in absence of beneficial bacteria. Probiotics can play a major role in modulating the intestinal and gut microbiome. *Lactobacillus acidophilus and L. casei shirota* are the most commonly used strains (Dasari et al., 2017; Maleki et al., 2016). Probiotics have the potential to inhibit the proliferation and growth of colorectal cancer (CRC) through a variety of methods, including the normalization of intestinal flora and the enhancement of the gastrointestinal barrier of the gastrointestinal tract. Short chain fatty acids (SCFAs), which are essential metabolites of probiotics, serve as a source of energy for the colonic mucosa, strengthen the intestinal protective barrier, regenerate the colonic epithelium, regulate the pH of the intestinal lumen, inhibit the proliferation of cancer cells, and encourage the death of cancer cells through the process of apoptosis (Wong and Yu, 2019). In addition, short-chain fatty acids (SCFAs) perform the function of signaling molecules through the use of G-protein coupled receptors (GPCRs), thereby reducing the production of pro-inflammatory cytokines and increasing the number of transgenic cells in the colon. There is still a lack of understanding regarding the mechanism of action of probiotics in the prevention and treatment of colorectal cancer (CRC) (Cristofori et al., 2021). As a result of the numerous forms of probiotics, which each have their own unique features and modes of action, the effects of these probiotics are complex and varied. It is required to do additional clinical research in order to examine the regulatory mechanisms of probiotics on colorectal cancer (CRC), understand each process, and then go on to use it as an adjuvant therapy for the prevention and treatment of CRC (Torres-Maravilla et al., 2021). Suitable probiotic-related treatments may be utilized for the purpose of preventing colorectal cancer (CRC) prior to the development of the disease (Ezeji et al., 2021). These interventions may include the direct oral delivery of probiotics and the fermentation byproducts of probiotics, in conjunction with probiotics or anticancer medicines. It is possible to use probiotics in conjunction with intensive cancer treatments such as surgery, chemotherapy, and immunotherapy in order to reduce the risk of complications during surgical and chemotherapeutic procedures, improve the effectiveness of chemotherapy, and enhance the quality of life of patients. Probiotics that are considered traditional are currently being used as an adjuvant therapy in the treatment and care of colorectal cancer (CRC), primarily for the purpose of mitigating surgical complications and alleviating the adverse effects of chemotherapy (Zhao et al., 2023).

### 6.2 Probiotics for treatment of GI tract issues

Probiotics can be effectively used for the treatment of lactose intolerance, gastrointestinal and urogenital infections, ulcerative colitis, gastrointestinal tumors and Chrohn’s disease. Probiotics compete with pathogens for the binding site at epithelial tissue and some synthesize biochemicals that inhibit the growth of pathogens. *Lactobacillus plantanum* effectively prevents bloating and abdominal pain, *S. boulardii* is used for the treatment of diarrhea and for improving overall gut functioning (Iannitti and Palmieri, 2010).

### 6.3 Probiotics for diabetes

Probiotics can effectively modulate gut hormones, the hormones are known for controlling homeostasis, their modification neutralizes the resistance to insulin which is the major cause of type 2 diabetes. There are some probiotics that can reduce the growth of adipocytes which aids in the prevention of a range of metabolic disorders (Westfall et al., 2018).

### 6.4 Probiotics for UTI

**Figure 2:**
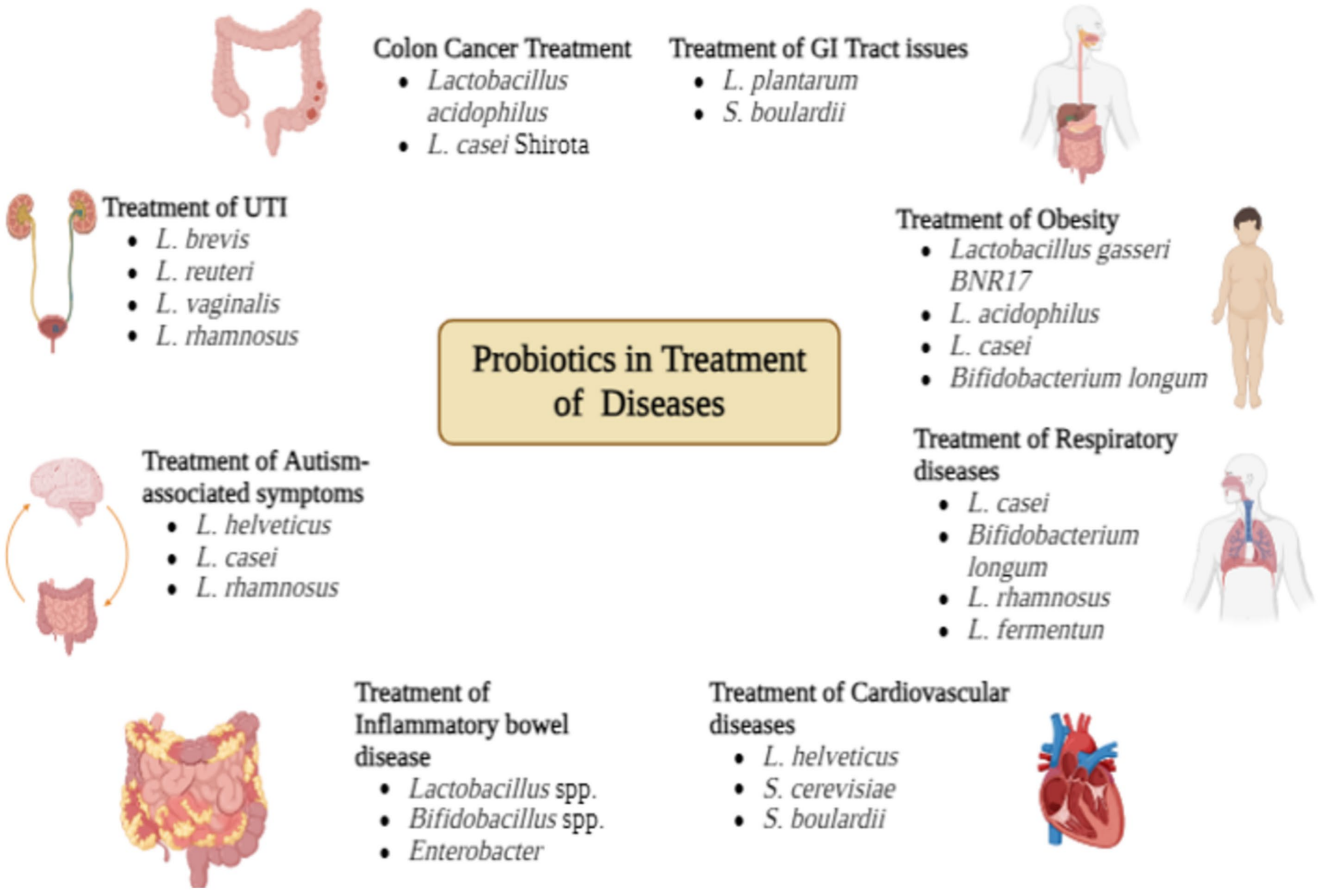
Probiotics in treatment of diseases.

Urinary Tract Infections (UTI) result from an imbalance in vaginal microbiota. It is quite common in both elder and young women. It can be both upper UTI and lower UTI. It is recurrent problem and prophylactic antibiotics can sometimes be beneficial but there is a risk of resistance development. Lactic acid bacteria have been found in the vaginal swab of many women are quite effective in lowering the pH. For treating UTI there are over 50 probiotics that can effectively treat UTI, all these are based on *Lactobacillus* spp. i.e. *Lactobacillus brevis*, *L. reuteri*, *L. vaginalis*, *L. rhamnosus* (Figure 2; Van et al., 2023).

### 6.5 Probiotics for obesity

Genetic variability, energy intake and expenditure imbalance are the major reasons for obesity which is a growing issue in these times. Adiponectin and leptin are present in adipocyte tissues and these are majorly responsible for obesity, *Lactobacillus gasseri* BNR17 inhibits their growth. Probiotics stimulate the adrenergic nervous system which generates a thermogenic response, this facilitates weight loss. Certain probiotics like *Lactobacillus acidophilus, L. casei, Bifidobacterium longum* exhibit hypocholesterolemic activity, they reduce the level of triglycerides, Low Density Lipids (LDL) and High Density Lipids (HDL) (George et al., 2018).

### 6.6 Probiotics for CNS and neurobiology

If *Lactobacillus plantanum* is administered to children with autism, it shows a promising effect. There are certain strains of *Lactobacillus* like *L. helveticus, L. casei, L. rhamnosus* which are known to reduce psychological distress, anxiety symptoms, autism-associated symptoms, respectively. In fact, some neuroactive compounds are being synthesized by some bacteria that resemble that of the host (Park et al., 2018). Probiotics and prebiotics affect the gut-brain axis, which bolsters the central nervous system and mitigates or regulates mental disorders such as depression, anxiety, autism, schizophrenia, and Alzheimer’s disease. This review presents intricate relationships among microbiota, the stomach, and the brain, along with recent research findings on the impact of probiotics and prebiotics on mental disorders. Probiotics and prebiotics may enhance central nervous system function and mitigate certain neurological illnesses. The relationship between the brain and the gastrointestinal tract is well-established (Victoria Obayomi et al., 2024). Mutual effects are ascribed to direct neural signals and indirect hormonal and enzymatic connections. A novel, natural therapy for mental disorders utilizing prebiotics, probiotics, and synbiotics to regulate the central nervous system with minimal side effects has been suggested. This review identified positive effects of prebiotics, probiotics, and synbiotics on anxiety, depression, stress, sleep, and Alzheimer’s disease (Centurion et al., 2022). Although research indicates the beneficial effects of pre-, pro-, and synbiotics on many mental disorders such as schizophrenia and autism spectrum disorder, the evidence is inadequate to justify their application for these conditions. It is essential to conduct meticulously organized clinical trials utilizing various prebiotics, probiotics, and synbiotics in clearly defined and sizable populations to obtain more precise and dependable results. Recent research is sufficiently persuasive to develop prebiotic, probiotic, and synbiotic formulations for mental disorders (Cryan et al., 2019). This may encompass the evaluation of pharmaceutical protocols alongside conventional treatments and the use of prebiotics, probiotics, or synbiotics (Ansari et al., 2023). Alterations in gut microbiota can influence mood, indicating that the microbiota–gut–brain (MGB) axis plays a role in depression. Numerous processes intersect with the role of gut bacteria in the development of metabolic disorders and obesity. In rats, prebiotics and probiotics modify the makeup and function of gut microbiota (Yuan et al., 2022). Probiotics and germ-free rodent models have demonstrated a causal relationship between microorganisms, microbial metabolites, and neurochemical signaling and inflammatory pathways in the brain. Further therapeutically pertinent research is required; nonetheless, probiotic supplementation has demonstrated modest antidepressant effects in individuals with depression (Franzosa et al., 2018). However, preclinical and clinical findings to critically assess the function of the MGB axis in the pathophysiology of depression and the putative communication pathways between the microbiota-gut interface and the brain. A comprehensive evaluation of methodologies in depression microbiome research is presented. Future MGB axis research must incorporate stringent placebo-controlled trials and a comprehensive molecular and biochemical understanding of prebiotic and probiotic mechanisms to translate preclinical successes into novel medicines (Radford-Smith and Anthony, 1880).

### 6.7 Probiotics for angiogenesis

New vessels are regenerated from old vessels via angiogenesis, it aids in faster wound healing. If not done in a proper way, it may lead to cancer and diabetic retinopathy. *S. boulardii* is known for protecting the host body against inflammation and injury by decreasing visceral hypersensitivity and modifying inflammatory cytokine profile (Xu et al., 2024).

### 6.8 Probiotics for respiratory diseases

Bronchitis, sinusitis, pharyngitis, rhinosinusitis, otitis is some of the most common respiratory disorders. Probiotics exhibit both anti-inflammatory and anti-microbial properties owing to which they can be used for the prevention of a number of respiratory disorders. For example: for controlling episodes of pneumonia in patients with cystic fibrosis, *Lactobacillus rhamnosus* can be administered. *Lactobacillus fermentum*, *L. casei*, and *Bifidobacterium longum* are some of the common probiotics used for the treatment of respiratory issues (Soccol et al., 2014).

### 6.9 Probiotics for cardiovascular diseases

Angiotensin-Converting Enzyme (ACE) is a key enzyme behind hypertension. *Lactobacillus helvetics and Saccharomyces cerevisiae* are known to synthesize peptides that can inhibit the activity of ACE (Mayta-Tovalino et al., 2023).

### 6.10 Probiotics for inflammatory diseases

Probiotics can be used for the treatment of Ulcerative colitis and Chrohn’s disease. They result in inflammation of GIT, both aerobic and anaerobic bacteria are responsible. These two diseases in combination are known as inflammatory bowel disease. *Lactobacillus*, *Enterobacter*, and *Bifidobacillus* are used for the treatment of inflammation (Roy et al., 2023).

## 7 Other recent approaches

Antimicrobials are quite effective but they do have limitations namely high cost, potential side effects and development of antimicrobial resistance with continued use (Vitor and Vale, 2011). In order to overcome this limitation antimicrobial drugs are being administered with probiotics, this not only improves the healing but also reduces the dose that is required to be administered, it ultimately leads to improved eradication of microbes from the site of infection and also reduces the potential side effects associated with full dosage (Kosgey et al., 2019). For the treatment of *Helicobacter pylori*-induced stomach ulcers antimicrobial are usually prescribed, they not only have potential side effects but they also develop antibiotic resistance in the causative organism. The eradication rate for an antibiotic is about 71% but when antibiotics are administered with probiotics this rate rises up to 81%. There is a significant reduction in side effects as well.

In 2018, Russo et al. studied the synergism of oral probiotic formulation and tropical clotrimazole for the treatment of vulvovaginal candidiasis. *Lactobacillus acidophilus* and *Lactobacillus rhamnosus* were used along with Lactoferrin glycoprotein. The combination of probiotics with clotrimazole effectively reduced the symptoms and it also affected the recurrence of infection (Shenoy and Gottlieb, 2019). Probiotics along with anti-fungal ointments are highly effective against *Candida* spp. they not only reduce the symptoms but also help in the restoration of normal vaginal microbiota (Mastubara et al., 2016).

Chronic periodontitis results from inflammation in tissues that support teeth, this leads to loss of periodontal ligament. It is a multifactorial disease; antibiotics are mostly used to reduce the bacterial load present. Probiotics can be used as adjuvant treatment along with conventional antibiotics. *L. reuteri* is especially used as it forms reuterin, it reduces oxidative stress and competes with pathogenic strains for adhesion sites, it also reduces the expression of MMP-8 by controlling the production of TNF-α, IL-17 which are pro-inflammatory cytokines (Soler and Kutsner, 2020).

## 8 Conclusion

This review focuses on Probiotics which is an active area of study and technology. It is an innovative topic for researchers. Numerous researches on probiotics indicated their potential applications in reducing the risk of diseases in some way. The use of probiotics expanded dramatically in recent years as a result of their successful delivery via various methods with minimal negative effects. Many probiotics have been approved for sale in large quantities to alleviate symptoms of diseases. The use of probiotics for the treatment of bowel disorders such as infectious diarrhea, antibiotic-induced diarrhea, lactose intolerance and allergies has been documented. The use of probiotics is not just limited to GIT disorders but has also expanded in the treatment of other disorders like obesity, respiratory, cardiovascular and CNS disorders. The delivery of probiotics to the site of action is another challenging task but there are various techniques available to overcome this limitation as well like the encapsulation of probiotic cells in a suitable carrier medium that is resistant to acids, bile salts and temperature so that the effect of probiotic can be maximized. Another approach to improve the effectiveness of probiotics is to administer them with antibiotics so that side effects can be reduced and the eradication of microbes can be enhanced without the development of antibiotic resistance.
